# United Against Rabies Forum: The One Health Concept at Work

**DOI:** 10.3389/fpubh.2022.854419

**Published:** 2022-04-13

**Authors:** Rachel Tidman, SM Thumbi, Ryan Wallace, Katinka de Balogh, Vivian Iwar, Isabelle Dieuzy-Labaye, Junxia Song, Sean Shadomy, Yu Qiu, Gregorio Torres, Jenny Hutchison, Bernadette Abela-Ridder, Katrin Bote, Sarah Beeching, Katy Cronin, Alexander Trees

**Affiliations:** ^1^World Organisation for Animal Health, Paris, France; ^2^Center for Epidemiological Modelling and Analysis, University of Nairobi, Nairobi, Kenya; ^3^Institute of Immunology and Infection Research, University of Edinburgh, Edinburgh, United Kingdom; ^4^Paul G. Allen School for Global Health, Washington State University, Pullman, WA, United States; ^5^Centers for Disease Control and Prevention, Atlanta, GA, United States; ^6^Food and Agriculture Organization of the United Nations, Rome, Italy; ^7^Economic Community of West African States Commission, Abuja, Nigeria; ^8^World Health Organization, Geneva, Switzerland; ^9^Oshun Partnership, London, United Kingdom; ^10^House of Lords, London, United Kingdom

**Keywords:** rabies, United Against Rabies, rabies elimination, *Zero by 30*, One Health, COVID-19, neglected tropical diseases, zoonosis

## Abstract

Human deaths from rabies are preventable and can be eliminated by applying a systematic One Health approach. However, this ancient disease still threatens the lives of millions of people in up to 150 countries and kills an estimated 59, 000 people every year. Rabies today is largely a disease of poverty, almost always linked to dog bites, with most deaths occurring in neglected communities in Africa and Asia. The disease places an immense economic burden on its victims, a cost that far outweighs the investment needed to control it. A global framework for rabies elimination in humans is set out in *Zero by 30: The Global Strategic Plan to end human deaths from dog-mediated rabies by 2030*. Despite the existence of proven control strategies and agreement on the path to eliminating human rabies deaths, mortality numbers from rabies remain high, and COVID-19 has set back efforts even further. But COVID-19 has also highlighted the value of a One Health approach to zoonotic disease and pandemic prevention. Rabies control programs offer a practical route to building One Health capacities that can also address other zoonotic threats, including those with pandemic potential. The United Against Rabies Forum aims to accelerate progress on rabies elimination while applying a One Health approach. The Forum promotes cross-sector collaboration among stakeholders and supports countries in their rabies elimination efforts. Increased political engagement and resource mobilization, both internationally and nationally, will be needed to achieve global rabies goals and can also make One Health implementation a reality.

## Introduction

Despite being entirely preventable, rabies threatens the lives of millions of people in up to 150 countries and kills an estimated 59, 000 people each year (1). The virus is transmitted through bites and scratches from infected animals, and while a variety of animals can host rabies, more than 95% of human rabies deaths are the result of infection from a rabid dog bite (2). Without prompt access to post-exposure prophylaxis (PEP), infection is fatal (2).

There is considerable global commitment to the elimination of dog-mediated rabies. In 2018, the Food and Agriculture Organization of the United Nations (FAO), World Organization for Animal Health (OIE), World Health Organisation (WHO) (the Tripartite) and the Global Alliance for Rabies Control (GARC) developed *Zero by 30: The Global Strategic Plan to end human deaths from dog-mediated rabies by 2030* (*Zero by 30*) (3). Central to this comprehensive strategy is a One Health approach which recognizes the intimate links between human, animal and environmental health, and promotes intersectoral collaboration to tackle public health challenges (3).

The COVID-19 pandemic has made One Health a policy priority in relation to future pandemic preparedness and the design of disease prevention and control strategies. The challenge is how to turn the One Health concept into practical action, especially in low-income settings. The current crisis has overwhelmed health systems and led to the redeployment of public health resources, resulting in setbacks to the control of endemic and neglected diseases such as rabies (4). However, rabies elimination can be a way to improve human and animal health system collaboration, boost engagement with communities and build workforce capacity, whilst delivering tangible results to the communities most vulnerable to rabies and other neglected diseases.

The United Against Rabies Forum (UAR Forum), an international One Health initiative, sets out to strengthen collaboration and coordination among partners, reduce fragmentation and coalesce cross-sector efforts, as well as supporting countries and regions to progress their rabies elimination efforts. Sustained political commitment and investment into such initiatives will not only end human deaths from rabies but will build One Health capacity to improve the response to other endemic and emerging infectious diseases, including those with pandemic potential.

## Rabies and One Health

The global pandemic has forced a major review of global health policy. One Health—based on the intimate links between human, animal and environmental health, has become a policy priority in designing disease prevention and control strategies and ensuring future pandemic preparedness. Rabies control is a model for One Health implementation, as its proven methodologies demonstrate the effectiveness of collaboration at the human-animal interface, including at community and municipal level. However, the pandemic has also set back many countries' efforts toward rabies elimination (4).

A recent survey found that rabies workforce capacity and financial resources were redirected toward COVID-19, movement restrictions limited access to healthcare facilities, and overwhelmed health systems had less capacity to treat cases of rabies exposure (4). Community outreach, including rabies prevention programs in schools, were suspended and the disruption of medical supply chains resulted in restricted PEP access for exposed individuals. The biggest impact on rabies control has been the cancellation or postponement of mass dog vaccination campaigns, a proven cost-effective strategy for saving human lives by stopping the transmission of rabies (3, 4). Rabies mainly affects the poorest communities, and these disruptions exacerbate existing inequalities, increasing the risk of death from rabies and imposing further health and financial burdens on communities already suffering from multiple endemic and neglected diseases (4).

Global policy makers are seeking ways to turn One Health approaches into practical action, including the establishment of the One Health High-Level Expert Panel (OHHLEP) (5). In this context, the elimination of rabies, for which there are effective human and animal vaccines and proven control strategies, can deliver concrete results for affected communities and build the integrated One Health systems that are urgently needed to protect us all (4). Successful rabies control programs require engagement across multiple ministries, public and private sectors, building intersectoral connections and networks which can also help to tackle other public health problems.

For example, Integrated Bite Case Management (IBCM) and contact tracing activities for rabies not only more accurately detect and treat rabies cases but create an active local reporting network that can improve surveillance for other diseases (6, 7). Building a One Health workforce capable of coordinated surveillance and response at community level builds integrated systems that can detect and respond to rabies as well as other zoonotic disease threats.

The burden of rabies is disproportionately borne by underprivileged communities, exacerbated by poor awareness and inequitable healthcare. Often the same health services in these communities are the point of contact for immediate treatment such as PEP, as well as for management of endemic and neglected diseases. Building and strengthening these connections between rabies and other neglected diseases using a One Health approach is likely to better use of existing networks, thus is more efficient, and likely to be more sustained. These are the workforces that have been redeployed during the COVID-19 pandemic (4, 8). The Zoonotic Disease Unit in Kenya is one example of a One Health framework that enhances communication pathways and builds coordinated responses to diseases such as rabies, while also improving systems for outbreak response (9). The One Health capacity-building required for rabies control will ensure that countries have a skilled workforce to respond to both new and existing public health challenges, while alleviating healthcare inequities and helping to break the cycle of neglect (9, 10).

Addressing rabies can also contribute to improved animal welfare, food security, healthier ecosystems and healthier cities. Rabies education and awareness help promote responsible dog ownership, humane dog population management and build more positive community relationships with dogs. There is a significant burden of livestock rabies on many pastoral cattle-keeping households that depend heavily on livestock for their livelihoods, with almost all cattle rabies attributed to rabid dogs in countries such as Bhutan and Ethiopia, and effective rabies control can improve food security by reducing livestock losses (11, 12). Vaccination in domestic canine reservoirs has been shown to effectively control rabies in threatened wildlife species such as African wild dogs and Ethiopian wolves, and a One Health approach which incorporates vaccination programs can contribute to conservation efforts (13). Rapid urbanization and urban slums have corresponded to an increase of waste production and poorly managed garbage disposal which can support free-roaming dog populations and increase the incidence of dog bites and rabies transmission (14, 15). This same issue is associated with other diseases, including dengue virus, leishmaniasis, Chagas disease and toxoplasmosis, and the improvement of waste management as part of an integrated rabies control program will also contribute to the control of other vector-borne and zoonotic diseases (16).

Rabies control strategies build connections across animal and human health systems and require engagement across multiple ministries and government sectors. The capacity-building required for rabies control will not only improve the health and livelihoods of millions of people in underserved and neglected areas, thereby building community resilience to the impacts of significant health events, but will also build health and surveillance systems to detect and prevent other endemic and emerging infectious diseases.

## United Against Rabies Forum: One Health in Action

A One Health approach is at the heart of the UAR Forum, which seeks to accelerate the sustained effort needed to deliver on the vision set out in *Zero by 30*. The UAR Forum was announced by the Directors-General of FAO, OIE and WHO (the Tripartite) in September 2020, building on the progress made during the implementation of Phase 1 (start up: 2018–2020) of *Zero by 30* (17).

**Figure 1:**
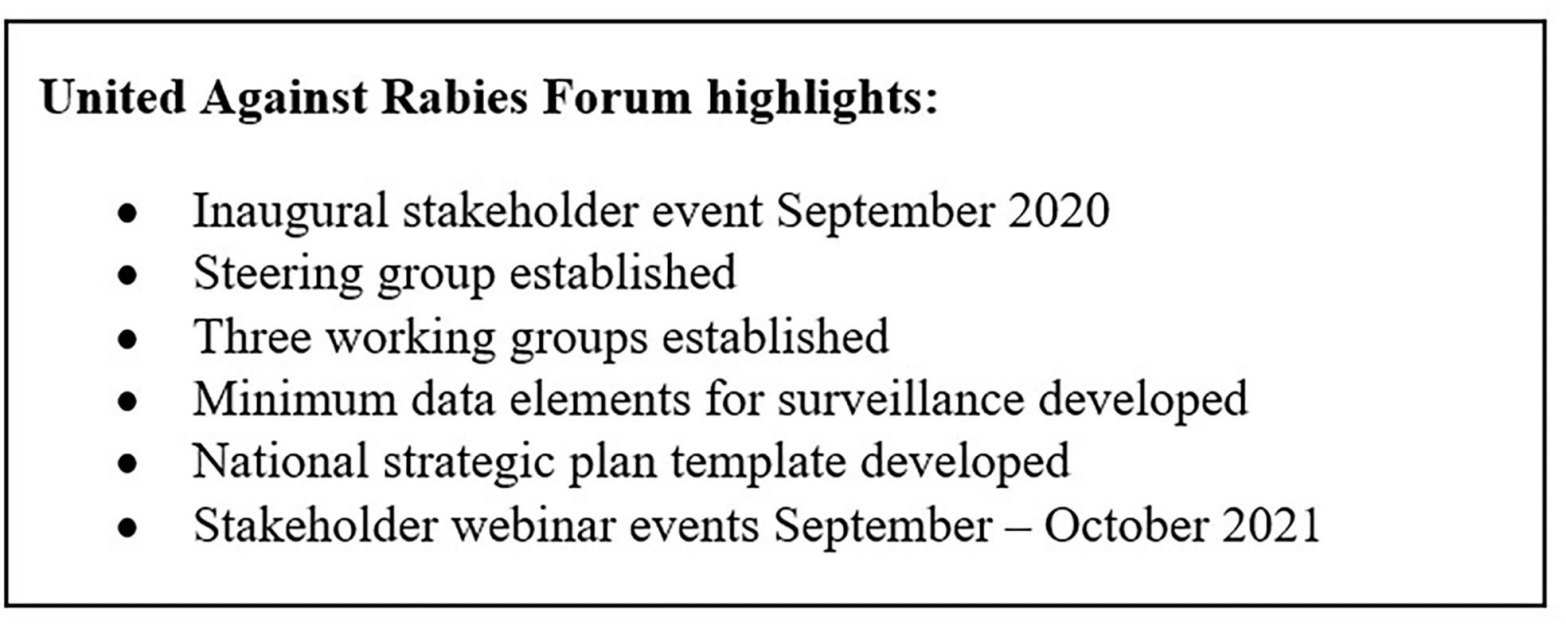
United Against Rabies Forum highlights.

Phase 2 (scale up: 2021–2025) plans to use the strong foundation established in Phase 1, refined and improved with learning and experience, to expand efforts and truly go global. The UAR Forum facilitates new and more inclusive ways to bring partners together across the world and provides a platform for rabies stakeholders to collectively work toward the goal of eliminating all human deaths from dog-mediated rabies. A key step has been the formation of results-focused working groups to progress priority activities which were defined by stakeholders in the UAR Forum Stakeholder meetings in 2020 and 2021 (18). Figure 1 shows the United Against Rabies highlights.

Working Group 1, is entitled “Effective use of vaccines, medicines, tools and technologies”. Initial work includes identifying minimum data elements to help countries strengthen their rabies surveillance systems and developing an evaluation process to help countries select and adapt existing tools for national control programs. This group will also map global rabies activities to facilitate coordination and assist stakeholders in assessing progress and identifying gaps. Other activities include exploring the benefits of dog identification and vaccination programs, and the review of evidence available for rapid diagnostic testing for rabies.

Working Group 2, entitled “Strategic and Operational Support” is focused on building One Health approaches and capacity, and promoting integrated country and regional strategies to progress rabies elimination. This group has developed a national strategic plan template to help countries develop their own rabies elimination plans and is progressing with the development of a monitoring and evaluation framework and a roadmap that provides countries with guidance and access to technical resources. Other priority activities being addressed by this group include identification of constraints that prevent countries from progressing toward elimination, revision of recommendations on oral rabies vaccines for dogs, and integration of rabies into other disease prevention and control programmes.

Working Group 3 is focused on “Resource Mobilization and Advocacy”. Priority activities for this working group include developing strategies and messaging for the engagement of national resource partners, engaging international resource partners to invest in rabies elimination as part of health system improvement, and creating a toolbox of advocacy and investment materials to engage public and private investors, media and civil society.

These working groups actively engage a wide range of stakeholders, representing national and regional bodies, the human and animal health sectors, NGOs, academic and research institutions, social science and private sectors. The UAR Forum encourages country leadership and facilitates access to technical expertise for sustained rabies control measures using a One Health approach (18).

The UAR Forum welcomes members from intergovernmental and non-government organizations, national governmental institutions, private sector entities, philanthropic foundations and academic and research institutions. The UAR Forum is free to join but asks member organizations to demonstrate support for the work, vision and purpose of the Forum, and be willing to actively share knowledge, information and innovations with other members to drive progress toward *Zero by 30* (18).

The UAR Forum website, which is in development, will provide a central platform where members and other stakeholders can access UAR Forum technical resources, while annual stakeholder events build the networks required to collectively overcome the challenges of achieving zero human deaths from dog-mediated rabies.

## Tripartite Initiatives

To complement this initiative, the Tripartite are working together to promote OIE's dog rabies vaccine bank and the creation of international standards to support rabies elimination efforts (19). The OIE vaccine bank facilitates the procurement of high-quality dog vaccines as a catalyst for mass dog vaccination campaigns. To access this, countries must show strong political will and a robust plan for implementing rabies control measures, and in alignment with the Tripartite's commitment to a collaborative One Health approach, WHO, FAO and other international agencies place procurement orders through the OIE rabies vaccine bank (18).

The Tripartite also encourages countries to apply for OIE endorsement of their national rabies control and elimination programs. This requires countries to adopt an efficient strategy in compliance with OIE International Standards, including intersectoral collaboration (19). Namibia and the Philippines were the first countries to receive OIE endorsement. The endorsements were made during the OIE 88th General Session in 2021 ensuring recognition of government engagement, facilitating access to international expertise and funding, and setting countries on a path toward rabies-free status (20).

OIE endorsement of national control programs may also support improved access to PEP via GAVI, the vaccines alliance. GAVI announced in 2019 that it would extend its portfolio to include human PEP (the vaccines administered if a person is suspected of being bitten by a rabid animal), but noted that this would need to be part of an integrated One Health approach, including the implementation of mass dog vaccination programs (21). However, the COVID-19 pandemic also highlighted challenges of equitable vaccine access, and in order for GAVI's decision to truly translate into improved PEP access for rabies-affected communities, the international community will need to support low-income and middle-income countries in building adequate platforms and infrastructure for vaccine delivery and access (22).

OIE and WHO is facilitating joint International Health Regulations Performance of Veterinary Services National Bridging Workshops (IHR-PVS NBW) with a specific focus on rabies, with the aim of enhancing cross-sector collaboration between human and animal health sectors (19). The OIE PPP Handbook provides guidelines for Public-Private Partnerships, promoting a joint approach to both sectors to work in synergy and share resources to improve outcomes for human and animal health (23). One Health was also identified as a priority area in FAO's new strategic framework launched in 2021, and FAO will further assist countries to strengthen their capacities to achieve the shared goals set out in *Zero by 30* (24).

The Tripartite is committed to using these activities to break down institutional silos and build One Health capacity to address rabies in a coordinated manner at country level. Importantly, the UAR Forum will support countries and other stakeholders by improving awareness of existing platforms and infrastructure, while providing the expertise required to help countries access these resources and progress their elimination efforts.

## One Health Resource Allocation

We know that control of rabies and the goal of eliminating human deaths from the disease requires sustained investment and political commitment both nationally and at local level. Unfortunately support for rabies elimination is often small-scale and fragmented, and competing priorities mean that stakeholders struggle to allocate sufficient resources to implement sustained rabies control measures (3). This is further compounded by the lack of high quality data on the incidence and socio-economic burden of rabies, without which countries are unable to prioritize rabies and advocate for political engagement and investment (3).

The One Health approach toward the elimination of human deaths from dog-mediated rabies needs to mean more than simply collaborating and sharing expertise across human and veterinary health sectors. There needs to be a systematic approach to One Health financing and integration of other underrepresented and non-traditional stakeholders, for example in tourism, environmental protection and education. The siloed nature of government budgets creates challenges in integrating diverse sectors to serve One Health objectives, however transdisciplinary approaches can reduce duplication, promote the sharing and pooling of resources and maximize the impact of investment. Promoting One Health resource allocation for rabies could create a model of integrated funding arrangements for other zoonoses and One Health needs.

In response to the pandemic, there has been growing commitment at the international level, for example in bringing together the OHHLEP—a valuable step in the right direction. However, One Health needs to be considered in the context of the burden of disease facing different countries and geographical regions. Therefore, national government buy-in is essential for the sustainability of both rabies control and other One Health programs.

Moreover, countries need to be empowered to drive their own progress toward the elimination of human deaths from dog-mediated rabies, prioritizing rabies at the highest political levels and allocating sufficient resources to support implementation of sustainable, One Health-informed, rabies control programs. For endemic countries, control of rabies could be considered a good proxy for the effectiveness of One Health collaboration, and a visible demonstration of commitment both to rabies elimination, and the building of robust national One Health programs. While international donors will inevitably support countries that can demonstrate their dedication to this cause, improving national self-financing, from either public or private sources including corporate foundations or philanthropy, will also work toward reduced international donor reliance.

Investment in rabies control is a money-saver. The cost of implementing effective national strategies to eliminate rabies is significantly lower than the estimated $US8.6 billion that rabies currently costs each year in expensive treatment and lives lost globally—a huge burden for the communities affected (1). The returns on investment in rabies control will be multiplied many times over if they are also made with a view to building One Health capacity to tackle other diseases, improving equity and access to care, and contributing to future pandemic preparedness.

## Conclusion

Strengthening efforts to eliminate human deaths from dog-mediated rabies offers an opportunity to put One Health into practice and make a significant contribution to health equity, stronger human and animal health systems, and future pandemic preparedness. The United Against Rabies Forum brings together a wide range of technical expertise and commitment to support countries' rabies control efforts and wider One Health implementation. To ensure these One Health approaches are effective and sustainable, the Forum will encourage members to collectively define priority activities and promote a shared responsibility of achieving the vision set out in *Zero by 30*. Focusing on a whole of society approach, the Forum will expand to engage more diverse stakeholders including local authorities and city leaders, social sciences, ecologists, conservationists and the wider NTD community. Political and financial commitment is needed but can deliver multiple returns while also meeting the goals set out in *Zero by 30*.

## Data Availability Statement

The original contributions presented in the study are included in the article/supplementary material, further inquiries can be directed to the corresponding author.

## Author Contributions

RT wrote the manuscript with input from all authors. All authors have reviewed and approved the manuscript.

## Funding

This work was supported by the World Organisation for Animal Health.

## Author Disclaimer

The authors alone are responsible for the views expressed in this article and they do not necessarily represent the views, decisions or policies of the institutions with which they are affiliated.

## Conflict of Interest

SB and KC were employed by Oshun Partnership. The remaining authors declare that the research was conducted in the absence of any commercial or financial relationships that could be construed as a potential conflict of interest.

## Publisher's Note

All claims expressed in this article are solely those of the authors and do not necessarily represent those of their affiliated organizations, or those of the publisher, the editors and the reviewers. Any product that may be evaluated in this article, or claim that may be made by its manufacturer, is not guaranteed or endorsed by the publisher.
